# Prescribing preoperative weight loss prior to major non-bariatric surgery for patients with elevated weight: a national provider survey (PREPARE provider survey)

**DOI:** 10.3389/fsurg.2025.1529116

**Published:** 2025-03-28

**Authors:** Tyler McKechnie, Alex Thabane, Phillip Staibano, Maisa Saddik, Olivia Kuszaj, Manon Guez, Dennis Hong, Aristithes Doumouras, Cagla Eskicioglu, Sameer Parpia, Mohit Bhandari

**Affiliations:** ^1^ Division of General Surgery, Department of Surgery, McMaster University, Hamilton, ON, Canada; ^2^ Department of Health Research Methods, Evidence, and Impact, McMaster University, Hamilton, ON, Canada; ^3^ Division of Otolaryngology Head and Neck Surgery, Department of Surgery, McMaster University, Hamilton, ON, Canada; ^4^ Division of General Surgery, Department of Surgery, St. Joseph Healthcare, Hamilton, ON, Canada; ^5^ Division of General Surgery, Department of Surgery, Centre Intégré Universitaire de Santé et de Service Sociaux de L’Est-de-L’Île-de-Montréal, Montréal, ON, Canada; ^6^ Department of Oncology, McMaster University, Hamilton, ON, Canada; ^7^ Division of Orthopedic Surgery, Department of Surgery, McMaster University, Hamilton, ON, Canada

**Keywords:** preoperative optimization, abdominal surgery, obesity, preoperative weight loss, very low energy diets

## Abstract

**Background:**

The surgical patient with obesity presents several challenges in intraoperative and postoperative care. We designed this cross-sectional survey to assess surgeon willingness to prescribe preoperative very low energy diets (VLEDs) and practice patterns in prescribing preoperative weight loss interventions for patients with obesity undergoing non-bariatric abdominal surgery.

**Methods and findings:**

We conducted a cross-sectional survey of practicing surgeons in Canada who perform major non-bariatric abdominal surgery, reported in accordance with the Consensus-Based Checklist for Reporting of Survey Studies and utilizing non-probability convenience sampling. The primary outcome was willingness to prescribe preoperative VLED to obese patients undergoing major non-bariatric abdominal surgery for both benign and malignant indications. We created a multivariable proportional odds model to identify factors associated with willingness to prescribe VLEDs. A total of 78 participants completed and returned the survey (response rate 10.9%; mean age 43.54 ± 8.13 years; 48.72% female). Most surgeons (79.5%) felt that obesity significantly impacted the technical difficulty of their operations. We identified a disconnect between those surgeons who were willing prescribe VLEDs vs. those who actually prescribed them (78.2% vs. 30.8%, respectively). Approximately half of the surgeons reported being unfamiliar with VLEDs. Regression analysis identified practicing in academic institutions was associated with increased willingness to prescribe [odds ratio (OR) 3.71, 95% confidence intervals (CI) 1.01–13.7, *p* < 0.01].

**Conclusion:**

Although the majority of surgeons feel that obesity adversely impacts perioperative care, only one-third routinely discuss preoperative VLEDs with their patients. Opportunities to increase awareness and evaluate the impact of VLEDs on patient outcomes remain high.

## Introduction

Obesity is an epidemic affecting upwards of 700 million people worldwide (1). Since 1985, the number of adults around the world living with obesity has risen by over 450% (2). With over 10% of Canadian children currently living with obesity, this number is set to continue growing (3). Thus, the surgical patient with obesity is becoming increasingly prevalent (4). This presents a significant challenge in major abdominal surgery, as operating in a field with large volumes of subcutaneous and visceral adiposity is difficult regardless of operative approach (5–7). For this reason, patients undergoing bariatric surgery are prescribed very low energy diets (VLEDs) for several weeks prior to their operation (8). These interventions are associated with reduced intraoperative difficulty and potentially a decrease in overall postoperative morbidity (9–12). Despite the advantages of VLEDs, they are infrequently used in non-bariatric abdominal surgery.

We recently conducted a systematic review aimed at identifying studies that evaluated the use of preoperative VLEDs for adult patients with obesity undergoing non-bariatric surgery (13). While the evidence was heterogenous, the available data suggests preoperative VLEDs are well tolerated, safe, and result in significant preoperative weight loss for patients with obesity (13). The included VLED protocols demonstrated excellent results, inducing between 3.2 kg and 19.2 kg of preoperative weight loss with near perfect adherence (94%–100%) (13). Given the heterogeneous and low quality evidence, we are in the process of designing a randomized controlled trial (RCT) aimed at conducting a high-quality, adequately powered assessment of the efficacy of VLEDs at reducing operative difficulty and improving postoperative outcomes for adult patients with obesity undergoing major non-bariatric surgery.

Prior to proceeding with an RCT, we were interested in assessing the willingness to prescribe preoperative VLEDs and other weight loss strategies. Currently, there are no published data describing surgeon willingness to prescribe preoperative weight loss interventions. Assessing willingness to prescribe will thus serve as crucial feasibility work for any RCTs aimed at assessing preoperative weight loss interventions (14). Therefore, we designed this national cross-sectional survey of practicing surgeons in Canada who perform major non-bariatric abdominal surgery with the objective of assessing current practice patterns of preoperative weight loss interventions for patients with obesity undergoing non-bariatric abdominal surgery. We hypothesized that a low proportion of practicing surgeons will be routinely prescribing preoperative VLEDs, but that willingness to prescribe will be high.

## Methods

### Study design

This was a national cross-sectional survey of practicing surgeons in Canada who perform major non-bariatric abdominal surgery. The study protocol has been published in *PLOS One* (15). This study was reported in accordance with the Consensus-Based Checklist for Reporting of Survey Studies (CROSS) (Supplementary Appendix S1) and received ethics approval from the Hamilton Integrated Research Ethics Board Ethics (Project #15775) (16). Written informed consent was obtained from all participants at the time of survey administration.

### Survey sample

The sampling frame was all independent, licensed, and practicing surgeons in Canada who perform major non-bariatric abdominal surgery. The overall sampling frame was estimated to be 716 surgeons (17–19). Surgical sub-specialties that were eligible for inclusion included general surgery, colorectal surgery, hepatobiliary surgery, surgical oncology, thoracic surgery, breast surgery, vascular surgery, urology, and gynecology. Surgeons who perform bariatric surgery exclusively were excluded.

### Sampling technique

We used a non-probability convenience sampling strategy to sample participants. We distributed an electronic survey to all surgeons that met inclusion criteria via email through provincial and national surgery associations (i.e., Canadian Association of General Surgeons, Ontario Association of General Surgeons, Association Québécoise de Chirurgie, Alberta Association of General Surgeons, Doctors of Manitoba, British Columbia Surgical Society, Canadian Collaborative on Urgent Care Surgery, Canadian Society of Surgery Oncology, Canadian Society of Colon and Rectum Surgeons) listservs between May 23rd, 2023 and August 23rd, 2023. We then contacted Department of Surgery administrators from all academic institutions across Canada to assist with further dissemination of the survey via institutional listservs between August 23rd, 2023 and November 23rd, 2023.

### Survey design

We designed the survey according to the Canadian Medical Association Journal *Guide for the Design and Conduct of Self-Administration Surveys for Clinicians* (20). A thorough literature review and consultation with content experts (one colorectal surgeon, one general surgeon, and two bariatric surgeons) informed survey questions. Prior to production of the final version of the survey, the methodology was critically appraised by two PhD biostatisticians. A bilingual member of the study team who is a practicing general surgeon in Montréal translated the survey into French. We piloted the survey with five local practicing surgeons prior to dissemination to the sample population. Pilot responses were not included in the final analyses.

The survey consisted of 31 items across five sections: (1) demographic information (e.g., age, sex, experience as independent surgeon, location of practice, type of practice); (2) institutional information (e.g., number of practicing surgeons, availability of bariatric surgery, availability of dieticians); (3) VLED practice patterns (e.g., willingness to prescribe, products used, types of surgeries in which preoperative VLEDs are useful); (4) VLEDs for oncology patients (e.g., willingness to prescribe, apprehension around prescribing); (5) free-text commentary. The full survey can be found in Supplementary Appendix S2.

### Survey administration

The survey was administered via an online form in RedCap®. Provincial and national surgical associations, as well as individual academic surgery departments were contacted twice during the sampling period and asked to disseminate the surveys to their respective audiences twice, spaced by four weeks (21). Reminder emails were sent at four-weeks to the associations and departments for re-distribution of the survey (22). Responses were collected for a total of five months following index distribution. Survey responses were anonymized according to chronological response number. If two survey responses were identical in terms of demographic information, they were assumed to be a result of multiple participation and the first complete survey response was used while other responses were removed from the dataset.

### Outcome measures

The primary outcome was the willingness to prescribe preoperative VLED to obese patients undergoing major non-bariatric abdominal surgery for both benign and malignant indications. This was measured using a five-point Likert scale (1—unwilling to consider; 2—mostly unwilling to consider; 3—likely willing to consider; 4—mostly willing to consider; 5—willing to consider). Secondary outcomes included: (1) frequency and type(s) of preoperative weight loss interventions currently being prescribed by practicing surgeons in Canada for obese patients undergoing non-bariatric abdominal surgery; (2) barriers to prescribing preoperative weight loss interventions; (3) factors associated with prescribing preoperative weight loss; (4) perceived benefits of prescribing preoperative weight loss; (5) knowledge surrounding preoperative weight loss options; (6) perceived difficulty of operating on obese patients for major abdominal surgery. Secondary outcomes were assessed using a combination of five-point Likert scale responses and narrative open responses.

### Sample size calculation

The sample size was calculated using methodology for determining survey sample size with a Likert scale primary outcome published by Park & Jung (23). The coefficient of variation was set at 0.3 (i.e., the standard deviation of responses will be half the value of the mean) given that respondents tend to avoid extreme responses in Likert scales. The pairwise correlation coefficient was set at 0.3 as the population is relatively heterogenous (i.e., surgeons practicing a variety of different surgical sub-specialties in a variety of different settings across Canada). The z-score associated with the accepted type I error is 1.96. The relative tolerable error was set at 5%. Given these assumptions and using sample size tables provided by Park & Jung, the minimum required sample size is 60.85, rounded to 61 (23).

### Statistical analysis

We performed all statistical analyses on Stata version 18 (StataCorp, College, TX) and created figures with Microsoft Excel©. We used descriptive statistics to describe the sample population. We presented continuous variables as means with standard deviation (SD) and ordinal variables as medians with interquartile ranges (IQR). Frequencies (n) and percentages (%) were used to characterize the data where appropriate. Likert scale responses were summarized as medians and IQR, analyzed as ordinal variables. To determine surgeon and institutional factors associated with the primary outcome, we created a multivariable proportional odds model. Resultant estimates were presented as odds ratios (OR) with 95% confidence intervals (CIs) for the following explanatory variables: sex, age, number of years of independent practice, location of practice, type of practice, surgical subspecialty, availability of a bariatric surgery center, availability of a dietician, and availability of a preoperative clinic run by a dedicated perioperative service or multidisciplinary team. Collinearity was assessed with the variance inflation factor (VIF). We performed *a priori* subgroup analyses of the primary outcome by the following subgroups: geographic location, type of center (i.e., rural, urban), type of practice (i.e., academic, non-academic), type of surgery, type of disease process (i.e., benign, malignant), and type of weight loss intervention. Respondents were classified as treating malignant diseases for the purpose of subgroup analyses if more than 75% of their case volume was estimated to comprise of oncologic cases. Narrative description of survey responses were provided and select written responses were collated, anonymized, and reported. Missing data was presumed to be missing at random and complete case analysis was performed.

## Results

### Respondent characteristics

A total of 78 participants completed and returned the survey (response rate 10.9% [i.e., 78/716]; mean age 43.54 ± 8.13 years, 48.72% female). Surgeon experience in years ranged from less than a year to 35 years (mean 11.43 ± 8.40 years). The majority of respondents practice in Ontario (*n* = 47, 60.3%). Half of the respondents were general surgeons (*n* = 39) and 19.2% were colorectal surgeons (*n* = 15). Most respondents had some proportion of cases within their practice that they were performing for oncologic disease (*n* = 72, 92.3%). Half of the respondents practiced in urban locations (*n* = 39) and nearly half worked in academic institutions (*n* = 36, 46.2%). Complete baseline respondent, practice, and institutional characteristics are found in Table 1.

**Table 1:** Survey respondent characteristics.

Characteristic	Overall *n* *=* *78*
Demographics	
Age, years [mean (SD)]	43.54 (8.13)
Female [*n* (%)]	38 (48.72)
Practice Characteristics	
Years in practice [mean (SD)]	11.43 (8.40)
Location of practice	
British Columbia [*n* (%)]	18 (23.08)
Alberta [*n* (%)]	5 (6.41)
Manitoba [*n* (%)]	1 (1.28)
Ontario [*n* (%)]	47 (60.26)
Quebec [*n* (%)]	5 (6.41)
Newfoundland and Labrador [*n* (%)]	2 (2.56)
Surgical specialty	
General surgery [*n* (%)]	39 (50.00)
Colorectal surgery [*n* (%)]	15 (19.23)
Hepatobiliary surgery [*n* (%)]	3 (3.85)
Surgical oncology [*n* (%)]	7 (8.97)
Foregut surgery [*n* (%)]	3 (3.85)
Thoracic surgery [*n* (%)]	2 (2.56)
Vascular surgery [*n* (%)]	2 (2.56)
Gynecology [*n* (%)]	3 (3.85)
Urology [*n* (%)]	3 (3.85)
Breast surgery [*n* (%)]	1 (1.28)
Oncology practice	2 (12.5)
Exclusively [*n* (%)]	1 (1.28)
>75% of cases [*n* (%)]	28 (35.90)
>25% of cases [*n* (%)]	43 (55.13)
None [*n* (%)]	6 (7.69)
Academic practice [*n* (%)]	36 (46.15)
Institutional Characteristics	
Urban location [*n* (%)]	39 (50.00)
Number of other surgeons	
0–2 [*n* (%)]	19 (24.36)
3–10 [*n* (%)]	53 (67.95)
>10 [*n* (%)]	6 (7.69)
Bariatric surgery available [*n* (%)]	27 (34.62)
Dietician available [*n* (%)]	67 (85.90)
Preoperative clinic available	
Anesthesia [*n* (%)]	40 (51.28)
Internal medicine [*n* (%)]	8 (10.26)
Perioperative medicine [*n* (%)]	12 (15.38)
Multi-disciplinary [*n* (%)]	9 (11.54)
None [*n* (%)]	9 (11.54)

*n*, number of respondents; SD, standard deviation.

### Perceived impact of obesity

**Figure 1:**
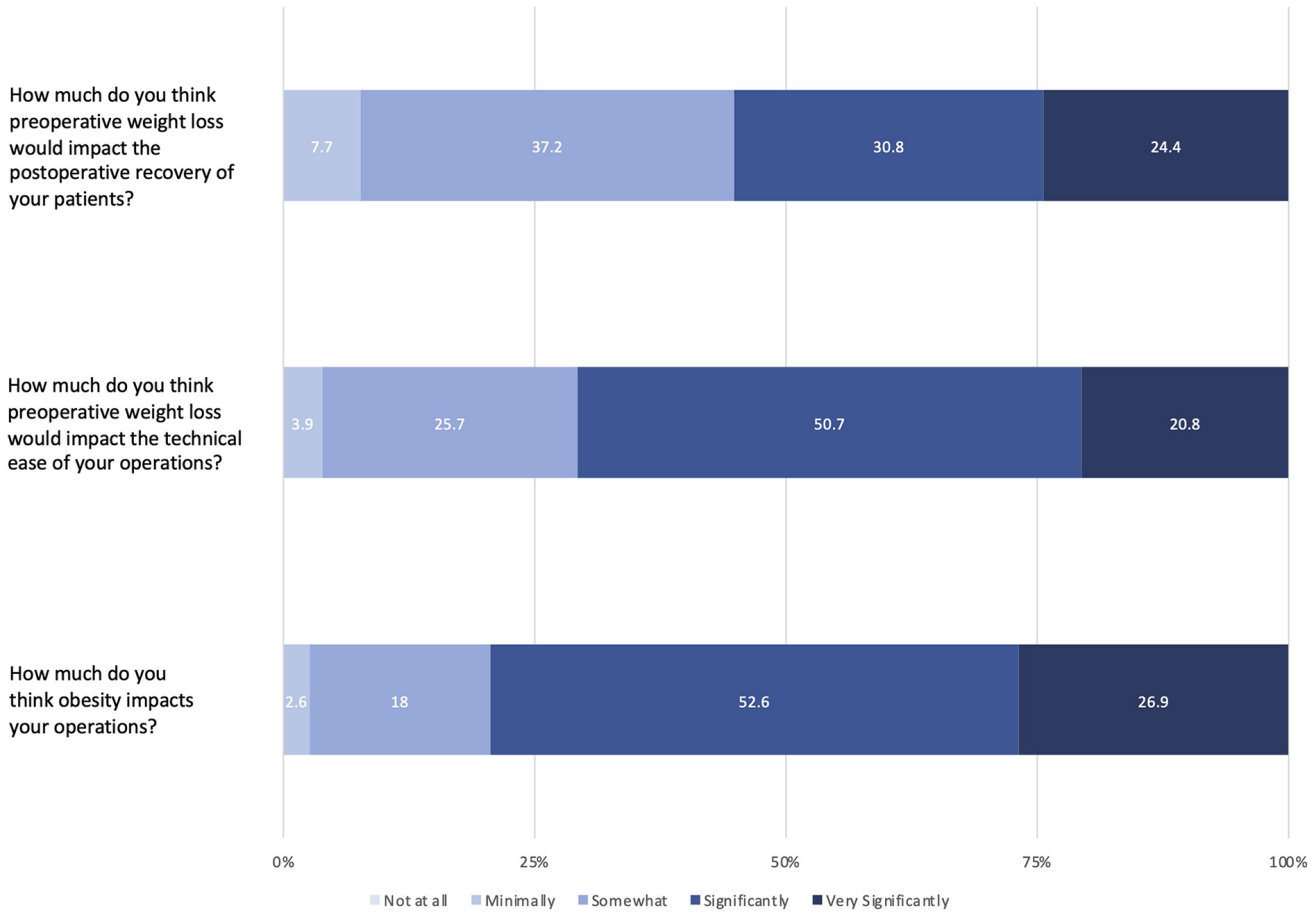
Likert-scale responses for the impact of obesity on perioperative outcomes.

Figure 1 demonstrates the surgeon responses to questions pertaining to the impact of obesity and preoperative weight loss on perioperative outcomes. Most surgeons felt that obesity significantly (52.6%) or very significantly (26.9%) impacted their operations. Most surgeons felt that preoperative weight loss impacted intraoperative technical ease and postoperative recovery significantly (50.7% and 30.8%, respectively) or very significantly (20.8% and 24.4%, respectively).

### Preoperative weight loss interventions

**Figure 2:**
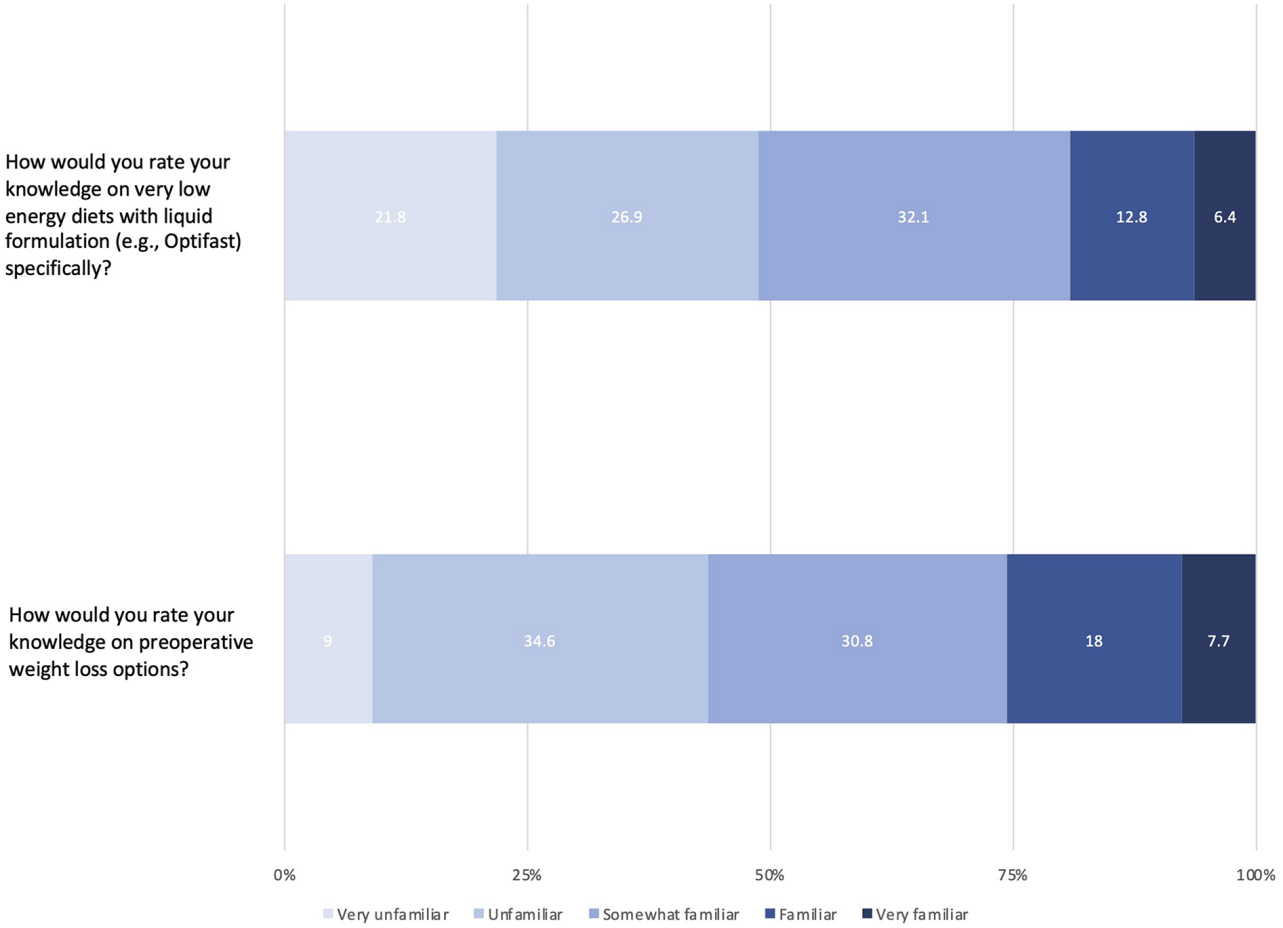
Likert-scale responses for self-perceived knowledge of preoperative weight loss options.

Table 2 describes current preoperative weight loss prescribing practices by respondents. Most surgeons only discuss preoperative weight loss with patients who have body mass indices (BMIs) greater than 35 kg/m^2^ (79.5%). For those patients above the stated BMI thresholds, 44.9% of surgeons stated that they sometimes discuss preoperative weight loss, 20.5% of surgeons stated that they almost always discuss preoperative weight loss, and 19.2% of surgeons stated that they always discuss preoperative weight loss. Three surgeons (3.8%) provided qualitative responses suggesting their discussions about preoperative weight loss with patients was “entirely dependent on the diagnosis and proposed surgery”. The most commonly discussed preoperative weight loss strategies were dietary modifications (78.2%) and exercise (69.2%), followed by VLEDs in liquid formulation (30.8%). Optifast was the most commonly prescribed VLED liquid formulation; the most common durations of VLED prescription were three (13.1%) and four (17.1%) weeks. Almost half the surgeons (48.7%) were unfamiliar with VLEDs in liquid formulation (Figure 2). Institutional barriers to the preoperative use of VLEDs that were cited as important included “cost” (18.0%), “product unavailability” (10.3%), and “lack of personnel to safely run and monitor preoperative weight loss programs” (12.8%).

**Table 2:** Preoperative weight loss prescribing practices.

Characteristic	Overall *n* *=* *78*
BMI cut-off for discussing preoperative weight loss	
>25 kg/m^2^ [*n* (%)]	1 (1.28)
>30 kg/m^2^ [*n* (%)]	15 (19.23)
>35 kg/m^2^ [*n* (%)]	27 (34.62)
>40 kg/m^2^ [*n* (%)]	21 (26.92)
Other [*n* (%)]	3 (3.85)
None [*n* (%)]	11 (14.10)
Frequency of discussing preoperative weight loss	
Never [*n* (%)]	2 (2.56)
Almost never [*n* (%)]	9 (11.54)
Sometimes [*n* (%)]	35 (44.87)
Almost always [*n* (%)]	16 (20.51)
Always [*n* (%)]	15 (19.23)
No response [*n* (%)]	1 (1.28)
Weight loss strategies discussed	
Exercise [*n* (%)]	54 (69.23)
Dietary modifications [*n* (%)]	61 (78.21)
VLED without liquid formula [*n* (%)]	2 (2.56)
VLED with liquid formula [*n* (%)]	24 (30.77)
Bariatric surgery [*n* (%)]	21 (26.92)
Weight loss clinic [*n* (%)]	22 (28.21)
Other [*n* (%)]	8 (10.26)
None [*n* (%)]	4 (5.13)
VLED liquid formula used	
Optifast [*n* (%)]	24 (30.77)
Modifast [*n* (%)]	4 (5.13)
Slimfast [*n* (%)]	4 (5.13)
Other [*n* (%)]	8 (10.26)
None [*n* (%)]	37 (47.44)
No response [*n* (%)]	1 (1.28)
VLED duration used	
1–2 weeks [*n* (%)]	1 (1.32)
3 weeks [*n* (%)]	10 (13.16)
4 weeks [*n* (%)]	13 (17.11)
5 weeks [*n* (%)]	6 (7.89)
6 + weeks [*n* (%)]	5 (6.58)
No response [*n* (%)]	41 (53.95)
Institutional barriers to prescribing VLED	
Yes [*n* (%)]	34 (43.59)
No [*n* (%)]	42 (53.85)
No response [*n* (%)]	2 (2.56)

*n*, number of patients; VLED, very low energy diet.

### Willingness to prescribe VLEDs

**Figure 3:**
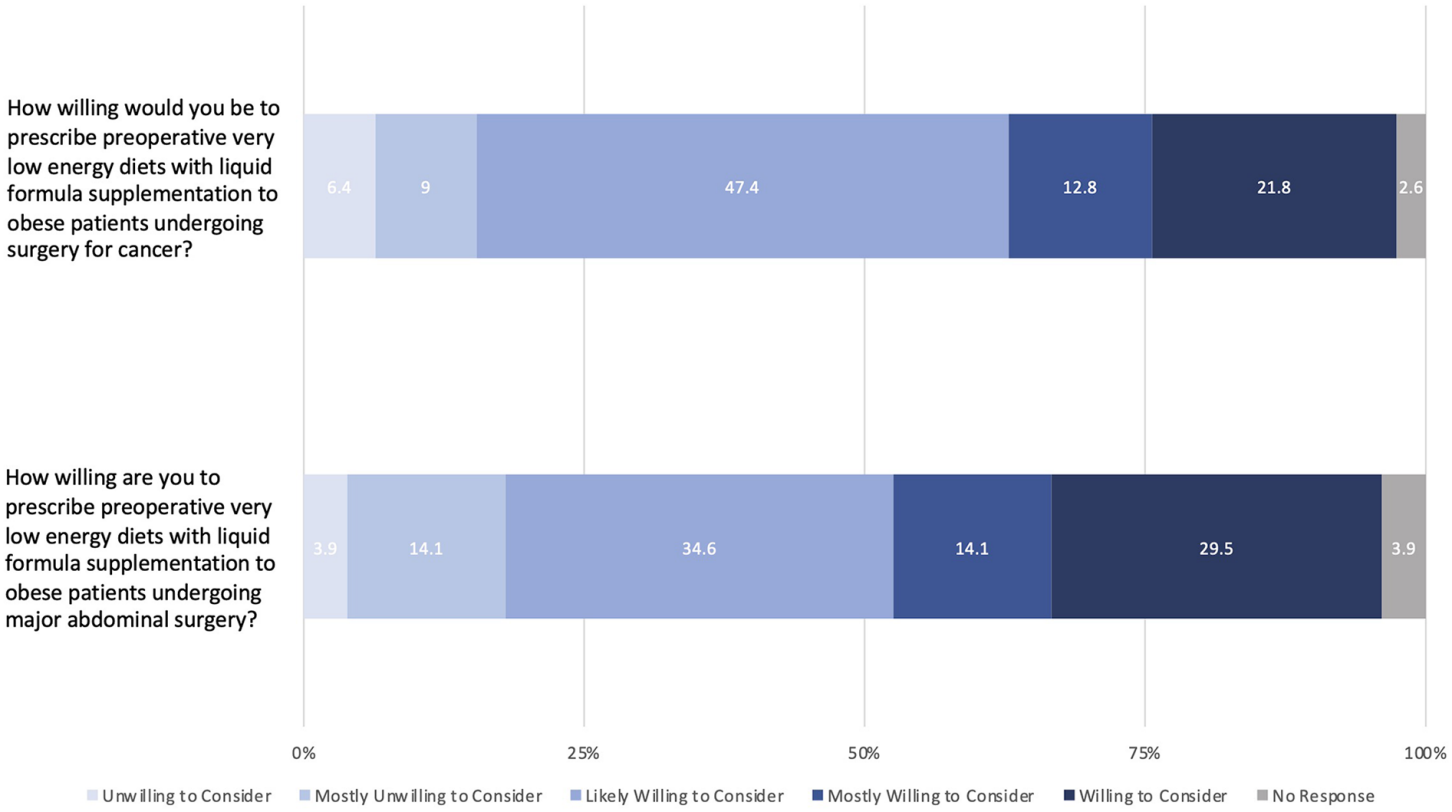
Likert-scale responses for willingness to prescribe preoperative very low energy diets.

**Figure 4:**
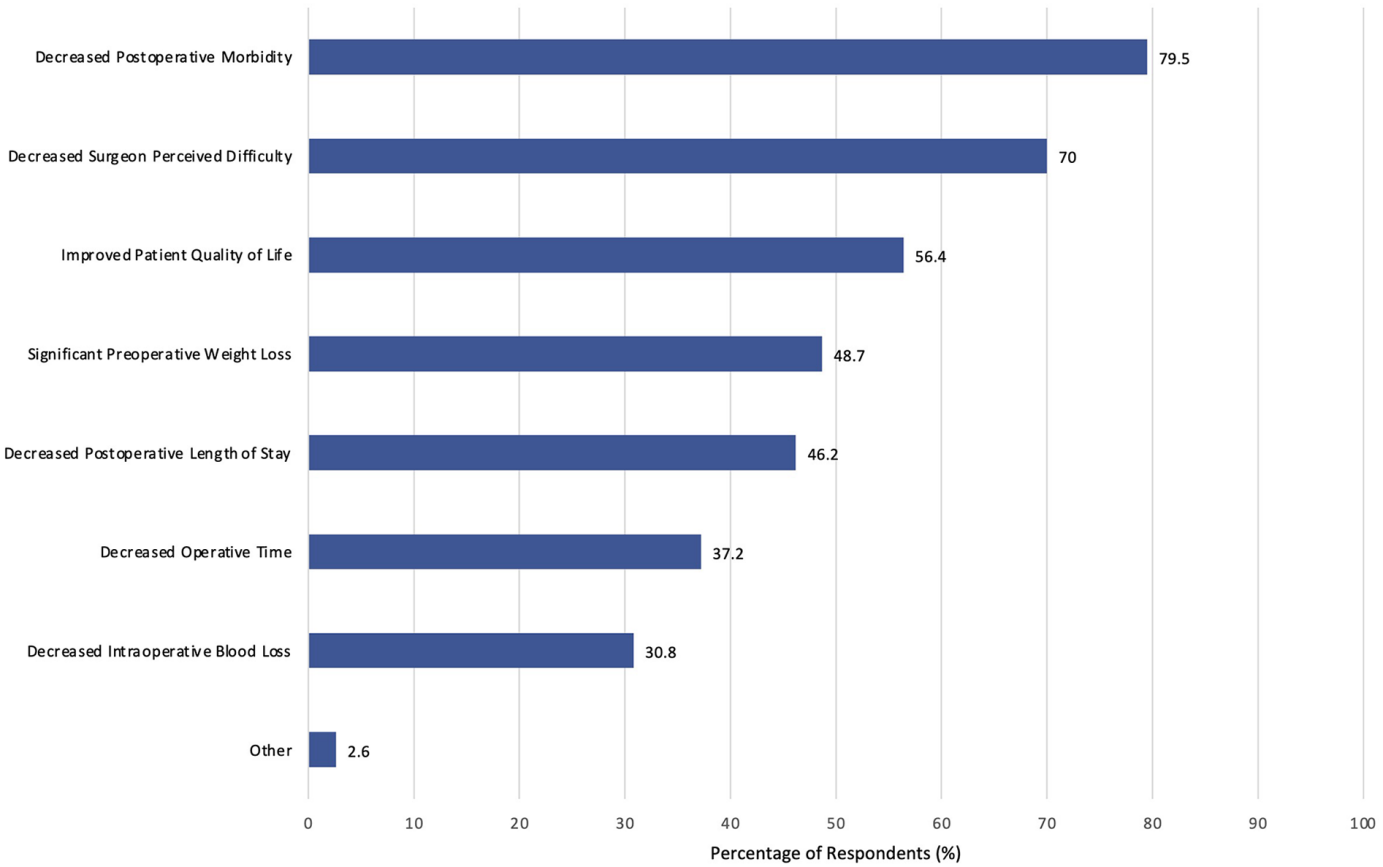
Proportion of respondents agreeing that the change in postoperative outcome would be clinically important.

Most surgeons were likely willing to consider (34.6%), mostly willing to consider (14.1%), or willing to consider (29.5%) prescribing preoperative VLEDs with liquid formulation to patients with obesity undergoing major abdominal surgery (Figure 3). Responses were similar when considering exclusively patients with obesity undergoing major abdominal surgery for cancer (likely willing to consider: 47.4%; mostly willing to consider: 12.8%; willing to consider: 21.8%). Respondents' rationales for outcomes associated with prescribing VLEDS included decreased postoperative morbidity (79.5%), decreased surgeon perceived technical difficulty (68.0%), and improved patient quality of life (56.4%). The full distribution of responses is found in Figure 4. Other surgeon responses included decreased incidence of surgical site infection, surgeon ergonomics, and long-term oncologic outcomes (e.g., overall survival, disease free survival). The majority of surgeon respondents are either not apprehensive (24.4%) or sometimes apprehensive (37.2%) about prescribing preoperative VLEDs to patients with cancer.

**Figure 5:**
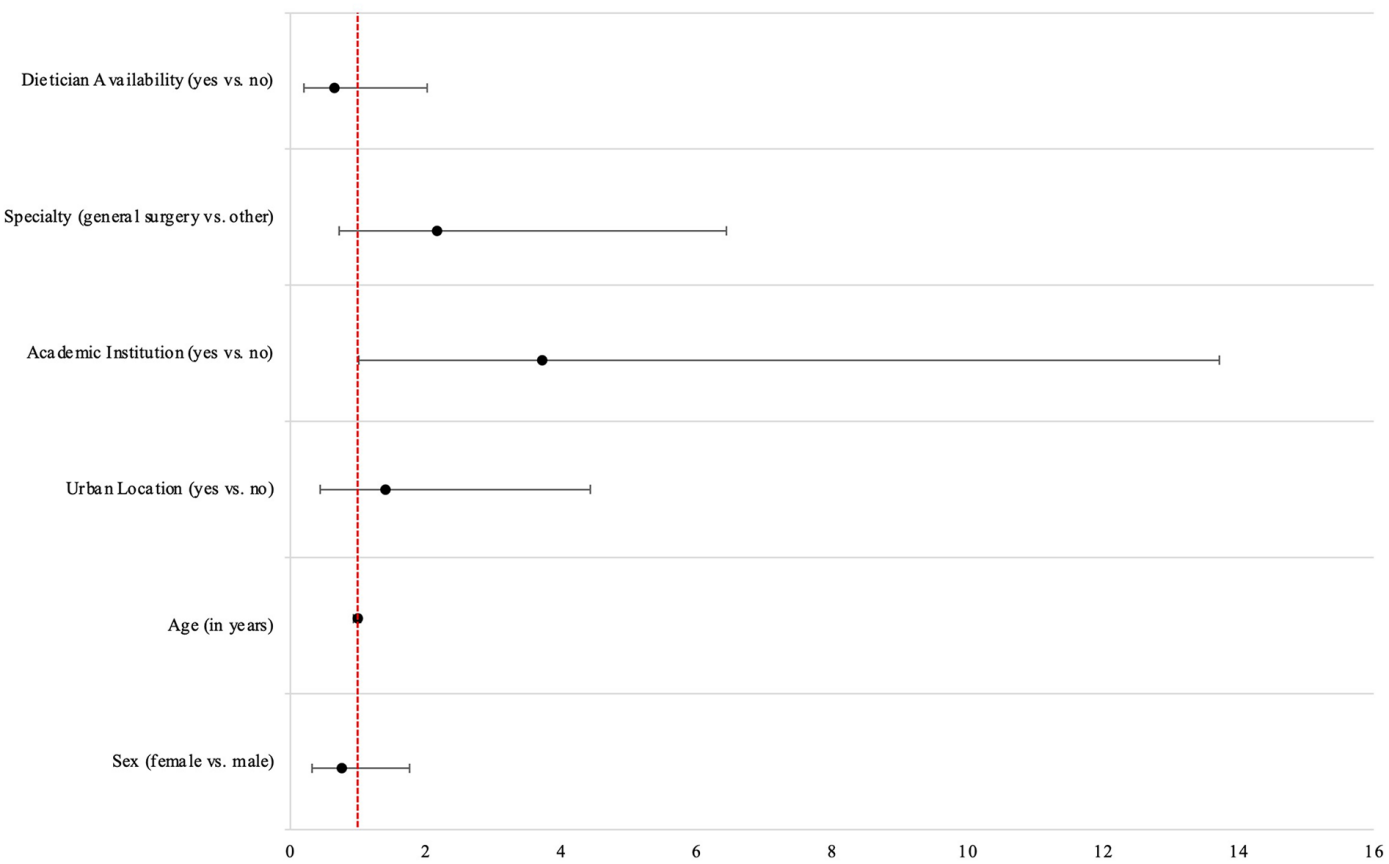
Odds ratios from multivariable proportional odds model for willingness to prescribe preoperative very low energy diets.

A proportional odds model was created to determine surgeon and institutional factors associated with willingness to prescribe preoperative VLEDs with liquid formulation. Experience in years and local availability of bariatric surgery, and local availability of multidisciplinary preoperative clinics were excluded from the final multivariable model, despite being included *a priori*, due to elevated VIF indicating collinearity. Being a surgeon at an academic institution was associated with increased willingness to prescribe (OR 3.71, 95% CI 1.01–13.72) (Table 3). All other baseline variables had 95%CIs crossing the line of no effect (Figure 5).

**Table 3:** Univariable and multivariable model for willingness to prescribe preoperative very low energy diets.

Variable	Unadjusted OR (95% CI)	Adjusted OR (95% CI)	*p*
Female (yes vs. no)	0.73 (0.32–1.65)	0.75 (0.32–1.76)	0.51
Age	1.00 (0.95–1.05)	0.99 (0.93–1.04)	0.68
Experience (in years)	1.00 (0.96–1.06)	–	–
Urban location (yes vs. no)	2.55 (1.09–5.96)	1.40 (0.44–4.43)	0.57
Academic institution (yes vs. no)	3.45 (1.44–8.28)	3.71 (1.01–13.72)	0.049*
Specialty (general surgery vs. other)	0.84 (0.37–1.92)	2.16 (0.73–6.44)	0.17
Bariatric surgery available (yes vs. no)	0.77 (0.32–1.87)	–	–
Dietician available (yes vs. no)	0.46 (0.16–1.38)	0.64 (0.20–2.02)	0.45
Preoperative clinic available (yes vs. no)	1.19 (0.88–1.59)	–	–

OR, odds ratio; CI, confidence interval.

* *p* *<* *0.05.*

### Subgroup analyses

Surgeons located outside of Ontario, at an urban care center, practicing in a surgical sub-specialty other than general surgery, practicing in an academic center, and with the majority of their practice focused on oncology had increased willingness to prescribe as compared to their counterparts (Table 4). Surgeons practicing in academic institutions were more willing to prescribe preoperative VLEDs with liquid formulation for patients undergoing surgery for cancer than surgeons practicing outside of academic institutions (Table 4).

**Table 4:** Likert-scale responses for subgroups.

Survey question	Unwilling to consider (1) [*n* (%)]	Mostly unwilling to consider (2) [*n* (%)]	Likely willing to consider (3) [*n* (%)]	Mostly willing to consider (4) [*n* (%)]	Definitely willing to consider (5) [*n* (%)]	No response [*n* (%)]	Median (IQR)
How willing are you to prescribe preoperative very low energy diets with liquid formula supplementation to obese patients undergoing major abdominal surgery?							
Overall (*n* = 78)	3 (3.85)	11 (14.10)	27 (34.62)	11 (14.10)	23 (29.49)	3 (3.85)	3 (3–5)
Location: Ontario							
Yes (*n* = 47)	1 (2.13)	8 (17.02)	17 (36.17)	2 (4.26)	17 (36.17)	2 (4.26)	3 (3–5)
No (*n* = 31)	2 (6.45)	3 (9.68)	10 (32.26)	9 (29.03)	6 (19.35)	1 (3.23)	3.5 (3–4)
Type of Center: Urban							
Yes (*n* = 39)	1 (2.56)	6 (15.38)	6 (15.38)	8 (20.51)	15 (38.46)	3 (7.69)	4 (3–5)
No (*n* = 39)	2 (5.13)	5 (12.82)	21 (53.85)	3 (7.69)	8 (20.51)	0	3 (3–4)
Type of Surgery: General surgery							
Yes (*n* = 39)	1 (2.56)	4 (10.26)	19 (48.72)	5 (12.82)	10 (25.64)	0	3 (3–5)
No (*n* = 39)	2 (5.13)	7 (17.95)	8 (20.51)	6 (15.38)	13 (33.33)	3 (7.69)	4 (2.5–5)
Type of Disease: Oncology							
Yes (*n* = 29)	2 (6.90)	4 (13.79)	6 (20.69)	5 (17.24)	11 (37.93)	1 (3.45)	4 (3–5)
No (*n* = 49)	1 (2.04)	7 (14.29)	21 (42.86)	6 (12.24)	12 (24.49)	2 (4.08)	3 (3–5)
Type of Practice: Academic							
Yes (*n* = 36)	1 (2.78)	4 (11.11)	7 (19.44)	6 (16.67)	16 (44.44)	2 (5.56)	4 (3–5)
No (*n* = 42)	2 (4.76)	7 (16.67)	20 (47.62)	5 (11.90)	7 (16.67)	1 (2.38)	3 (3–4)
How willing would you be to prescribe preoperative very low energy diets with liquid formula supplementation to obese patients undergoing surgery for cancer?							
Overall (*n* = 78)	5 (6.41)	7 (8.97)	37 (47.44)	10 (12.82)	17 (21.79)	2 (2.56)	3 (3–4)
Location: Ontario							
Yes (*n* = 47)	3 (6.67)	5 (10.64)	19 (40.43)	6 (12.77)	12 (25.53)	2 (4.26)	3 (3–5)
No (*n* = 31)	2 (6.45)	2 (6.45)	18 (58.06)	4 (12.90)	5 (16.13)	0	3 (3–4)
Type of Center: Urban							
Yes (*n* = 39)	1 (2.56)	4 (10.26)	17 (43.59)	4 (10.26)	13 (33.33)	0	3 (3–4)
No (*n* = 39)	4 (10.26)	3 (7.69)	20 (51.28)	6 (15.38)	4 (10.26)	2 (5.13)	3 (3–4)
Type of Surgery: General surgery							
Yes (*n* = 39)	3 (7.69)	4 (10.26)	19 (48.72)	7 (17.95)	6 (15.38)	0	3 (3–4)
No (*n* = 39)	2	3	18	3	11	2	3 (3–5)
Type of Disease: Oncology							
Yes (*n* = 29)	1 (3.4)	1 (3.4)	14 (48.28)	1 (3.4)	12 (41.38)	0	3 (3–5)
No (*n* = 49)	4 (8.16)	6 (12.24)	23 (46.94)	9 (18.37)	5 (10.20)	2 (4.08)	3 (3–4)
Type of Practice: Academic							
Yes (*n* = 36)	0	3 (8.33)	14 (38.89)	4 (11.11)	13 (36.11)	2 (5.56)	3.5 (3–5)
No (*n* = 42)	5 (11.90)	4 (9.52)	22 (52.38)	6 (14.29)	4 (9.52)	1 (2.38)	3 (3–3)

*n*, number of respondents; IQR, interquartile range.

## Discussion

Surgeons across Canada demonstrated a strong willingness to prescribe preoperative VLEDs to patients undergoing major intra-abdominal surgery for both benign and malignant disease. Over 75% of surgeons stated they would be willing to consider the use of preoperative VLEDs for patients with obesity undergoing major abdominal surgery, yet only 30% routinely discuss preoperative VLEDs with their patients currently.

Obesity represents a substantial challenge in the context of intra-abdominal surgery (24). The increased adiposity and altered anatomy associated with obesity pose technical difficulties for surgeons, complicating tasks such as exposure, dissection, and closure (25). Postoperative outcomes in individuals with obesity frequently include higher rates of wound infections, respiratory complications, and even death (24, 26). As such, the management of intraabdominal surgery in obese patients necessitates a comprehensive understanding of the associated challenges and a tailored approach to optimize perioperative care and mitigate postoperative risks. The findings of the present survey indicate that the current Canadian surgical work force understands the risks associated with obesity and acknowledges the impact on both intraoperative and postoperative outcomes. Over three-quarters of the surgeons sampled in this survey felt obesity significantly impacted the operations they perform. It naturally follows that several weight loss strategies prior to surgery have been proposed and used in clinical practice. Among the most commonly used according to this survey were dietary modifications (78.2%) and exercise (69.2%).

The value of weight loss as an essential component of preoperative optimization for patients with obesity undergoing non-bariatric abdominal surgery is clearly recognized by the contemporary Canadian surgeon. Over 70% of surgeons surveyed agree that preoperative weight loss increases the technical ease of their operation and over 50% agree that preoperative weight loss improves postoperative recovery. Yet only 39.7% of the surgeons surveyed always or almost always discuss preoperative weight loss with their patients. Moreover, while easily implemented interventions such as dieting and exercise are discussed frequently, novel and more effective interventions such as VLEDs and bariatric surgery are not commonly discussed (13). It must be noted that for lifestyle-based interventions, such as VLEDs and physical activity, long-term efficacy can be limited (27, 28). Furthermore, recent epigenetic investigations of adipocytes in mouse models highlight the long-term persistence of obesogenic memory following weight loss (29). While bariatric surgery may address these shortcomings, as the most effective method for sustained weight loss and metabolic recovery, it can be difficult to implement preoperatively, especially in the setting of surgery for oncologic disease (30, 31). Thus, while bariatric surgery offers benefits in terms of efficacy, VLEDs are likely a more practical intervention in this setting (9, 30, 31). Despite most surgeons being willing to prescribe preoperative VLEDs to their patients, nearly half of the surveyed surgeons stated that they were very unfamiliar or unfamiliar with preoperative VLED prescription.

The lack of familiarity with a specific intervention can significantly impact a physician's confidence and comfort in prescribing it to their patients (32). This hesitancy may arise from a dearth of knowledge regarding the intervention's mechanisms, efficacy, and safety profile (32). Medical professionals are more likely to embrace interventions with which they are well-acquainted, as familiarity fosters a sense of trust and understanding. As highlighted by the findings of this survey, surgeons across Canada are not routinely prescribing preoperative VLEDs for patients undergoing non-bariatric surgery, perhaps because of unfamiliarity. Surgeons at academic institutions, which are more likely to have bariatric surgery programs, are more willing to prescribe this intervention. This likely stems, at least in part, from proximity to these bariatric programs in which preoperative VLEDs are standard of preoperative care. There is RCT level evidence supporting the use of preoperative VLEDs in bariatric surgery (10–12). VLEDs may reduce postoperative LOS by as much as a full day, decrease visceral fat by as much as 29%, and perhaps may even decrease overall postoperative morbidity by as much as 33% (9–12). A similar level of evidence does not exist for non-bariatric abdominal surgery. There have been four RCTs evaluating preoperative VLEDs in non-bariatric surgery, all of which are at high risk of bias and the largest of which included 76 patients (33–36). This highlights the need for well-designed, adequately powered RCTs evaluating this intervention in the non-bariatric surgery population. With a more solid foundation of evidence supporting the use of preoperative VLEDs in non-bariatric abdominal surgery, surgeons may feel more comfortable prescribing this intervention, and do so on a regular basis. There are likely other important factors to consider in terms of willingness to prescribe that would need to be addressed in the future, including cost, availability of VLEDs, and personnel available for monitoring preoperative weight loss interventions.

The acceptance and adoption of a novel medical intervention by healthcare providers is contingent upon their willingness to incorporate it into their existing therapeutic repertoire. Thus, the evaluation of clinicians' willingness to prescribe a medical intervention is pivotal for the success of a VLED trial, as well as the effective implementation of the intervention into clinical practice (37, 38). Understanding clinicians’ perspectives, concerns, and motivations related to prescribing a specific intervention is essential for identifying potential barriers and facilitators to its adoption (37). Research that incorporates such assessments can inform tailored implementation strategies, enhancing the likelihood of successful integration into routine clinical care. The current survey serves as critical feasibility and exploratory research as we design a definitive RCT aimed at evaluating preoperative VLEDs for major non-bariatric surgery. In particular, this work provides valuable insight into the types of outcomes that would convince surgeons and policy makers that this is a worthwhile intervention for preoperative optimization of patients with obesity. Over 70% of surgeons responded that evidence demonstrating preoperative VLEDs can decrease postoperative morbidity and surgeon perceived technical difficulty in the operating room would increase their willingness to prescribe this intervention. As such, future RCTs evaluating this intervention, and all other preoperative optimization techniques in patients with obesity, should aim to focus on these as primary outcomes.

Our study has several strengths including its strict adherence to reporting guidelines and survey creation best practices, meeting the *a priori* sample size calculation, the collection of both quantitative and qualitative data, administration in both English and French, and the use of regression analyses to identify factors associated with increased willingness to prescribe preoperative VLEDs. There are, however, several limitations to consider when interpreting these findings. First, this study is at risk of sampling bias. Specifically, the use of non-probability convenience sampling puts these data at risk of selection bias and decreases generalizability (39). Low response rates in surveys administered to healthcare practitioners are a widely recognized limitation in survey-based research and can be due to various factors such as time constraints, survey burden, and competing priorities (40). We attempted to mitigate this through a long sampling period (i.e., five months) to increase the number of responses received. Secondly, these survey data may also be limited by under coverage bias as we only sampled a small proportion of Canadian surgeons performing intra-abdominal surgery. Moreover, our sample was mostly derived from general surgeons and colorectal surgeons from British Columbia and Ontario. Thirdly, while the sample size of the present survey provided robust Likert scale data pertaining to willingness to prescribe preoperative VLEDs, the proportional odds model was underpowered, as evidenced by the wide 95%CIs associated with our point estimates. The resultant risk of type II error thus limits our confidence in the estimated association of these covariates with willingness to prescribe preoperative VLEDs. Lastly, this is a cross-sectional study which places these data at increased risk of residual confounding bias (41).

## Conclusion

This national survey of major non-bariatric surgeons found that most surgeons are unfamiliar with VLEDs and do not use them frequently but are willing to consider prescription of preoperative VLEDs for preoperative optimization of patients with obesity. They highlighted that evidence demonstrating decreased postoperative morbidity and intraoperative technical difficulty would contribute to their willingness to use these interventions routinely. These data will serve as crucial background work for a definitive RCT evaluating the efficacy of preoperative VLEDs for patients with obesity undergoing major non-bariatric surgery.

## Data Availability

The original contributions presented in the study are included in the article/Supplementary Material, further inquiries can be directed to the corresponding author.
